# The viscosity of atmospherically relevant organic particles

**DOI:** 10.1038/s41467-018-03027-z

**Published:** 2018-03-06

**Authors:** Jonathan P. Reid, Allan K. Bertram, David O. Topping, Alexander Laskin, Scot T. Martin, Markus D. Petters, Francis D. Pope, Grazia Rovelli

**Affiliations:** 1 School of Chemistry, University of Bristol, Manchester, BS8 1TS UK; 2 Department of Chemistry, University of British Columbia, Vancouver, BC Canada; 3 School of Earth, Atmospheric and Environmental Science, University of Manchester, Manchester, M13 9PL UK; 4 Department of Chemistry, Purdue University, West Lafayette, IN USA; 5 School of Engineering and Applied Sciences, Harvard University, Cambridge, MA 02138 USA; 6 Department of Earth and Planetary Sciences, Harvard University, Cambridge, MA 02138 USA; 7 Department of Marine Earth and Atmospheric Sciences, North Carolina State University, Raleigh, NC 27695 USA; 8 School of Geography, Earth and Environmental Sciences, University of Birmingham Edgbaston, Birmingham, B15 2TT UK

## Abstract

The importance of organic aerosol particles in the environment has been long established, influencing cloud formation and lifetime, absorbing and scattering sunlight, affecting atmospheric composition and impacting on human health. Conventionally, ambient organic particles were considered to exist as liquids. Recent observations in field measurements and studies in the laboratory suggest that they may instead exist as highly viscous semi-solids or amorphous glassy solids under certain conditions, with important implications for atmospheric chemistry, climate and air quality. This review explores our understanding of aerosol particle phase, particularly as identified by measurements of the viscosity of organic particles, and the atmospheric implications of phase state.

## Abstract (web summary)

The phase state of organic particles in the atmosphere has important consequences for the impact of aerosols on climate, visibility, air quality and health. Here, the authors review the evidence for the formation of amorphous glassy particles and the methods for determining aerosol particle viscosity.

## Introduction

Atmospheric particles, which range in size from <10 nm to ∼10 μm, consist of both inorganic and organic material^1,2^. The organic component can represent 50% or more of the mass of the fine aerosol particle fraction (particles smaller than 1 μm in diameter) in the atmosphere^2^. Ambient organic aerosols (OA) can be emitted directly (referred to as primary OA) or can be formed by a complex series of reactions (referred to as secondary OA, SOA)^3^. The composition of SOA is especially uncertain, with only approximately 10% of the mass of secondary OA identified at the molecular level^2^. Until recently, researchers assumed that atmospheric organic particles are liquid in phase and, hence, have low viscosity. In stark contrast, recent measurements of organic particle properties often imply the existence of highly viscous semi-solid and even amorphous solid particles^4–6^. Our focus here is to explore the compositional and environmental factors that govern particle viscosity, the consequences of particle viscosity for aerosol microphysics and the global impacts that can then result. Many of the studies on which our understanding of atmospheric aerosol particle viscosity is based have been undertaken in the laboratory using surrogates of ambient particles; this is a consequence of the challenges of making aerosol particle viscosity measurements directly. Thus, much of our review necessarily considers the consensus that is emerging from laboratory work on the phase state of ambient particles, supported by the limited number of field measurements that are now emerging.

**Fig. 1:**
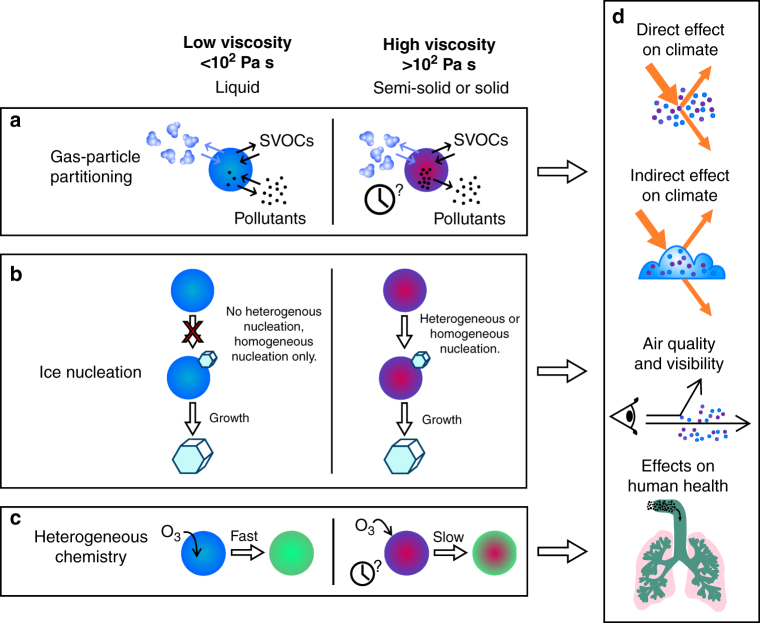
Impacts of ambient particle viscosity and phase on climate and health. **a** Although low-viscosity aerosol particles equilibrate in composition rapidly, highly viscous particles may require time to achieve an equilibrium composition through the gas-particle partitioning of water, semi-volatile organic compounds (SVOCs) and other pollutants. **b** Unlike liquid droplets, glassy particles can act as heterogeneous nuclei for ice nucleation. **c** Heterogeneous chemistry can occur rapidly in low-viscosity particles throughout the particle bulk. In viscous particles, heterogeneous chemistry may occur only very slowly and be confined to the particle surface. **d** In combination, differences in the mechanisms and rates of microphysical processing in viscous aerosol particles when compared to low-viscosity solution droplets can have important consequences for the impacts of aerosols on climate, visibility, air quality and health

The viscosity of atmospheric OA is central to rationalising and predicting their atmospheric impacts (Fig. 1)^7–10^. As an example, the rates of growth and evaporation of organic particles are dependent on particle viscosity, with direct implications for climate, visibility and air quality^10–12^. Liquid organic particles with low viscosity are responsive to changes in gas-phase composition and take up or lose water in response to variations in ambient relative humidity (RH) and temperature^13,14^. By contrast, highly viscous organic particles can be slow to respond to changes in gas-phase composition, particularly under dry conditions or at low temperatures^5,7,13^. Einstein first established the slowing of diffusional motion in a medium of increasing viscosity, with this inverse relationship apparent in the Stokes–Einstein equation:

1 $$\eta = \frac{{k_{\mathrm B}T}}{{6\pi Dr}},$$

where *η* is the dynamic viscosity of the fluid (Pa s), *k*_B_ is the Boltzmann constant (J K^−1^), *T* is temperature (K), *D* is the diffusion constant (m^2^ s^−1^) of the diffusing molecule (or particle) of radius *r*^15^. The Stokes–Einstein equation suggests that an increase of 15 orders of magnitude in viscosity, as might be anticipated if a particle transitions from a dilute aqueous solution to an amorphous glass, could lead to a concomitant reduction in the diffusion constant of molecular species. Although direct measurements of diffusion constants in atmospheric aerosol particle surrogates are desirable, very few measurements currently exist and there are no measurements in actual ambient particles; instead, measurements of viscosity and the application of the Stokes–Einstein equation is the only route available for estimating diffusion constants^8,10,11^.

The dramatic decrease in diffusion constant that can be expected to accompany an increase in viscosity can lead to a lengthening in the diffusional mixing time, *τ*_mix_, within a particle of radius *a*, which can be estimated from^16^:

2 $$\tau _{{\mathrm mix}} = \frac{{a^2}}{{\pi ^2D}}.$$

Such a lengthening has been used to propose significant kinetic limitations on growth and evaporation (Fig. 1a). Indeed, single particle models initially suggested that mixing timescales could increase to anywhere from >1 h to 1 year depending on the particle size^17^. More realistic treatments of varying particle viscosity with changing RH indicate these timescales could decrease to micro-seconds at high RH due to the plasticising effect of water^18^. This level of sensitivity to moisture content is confirmed experimentally in single particle^16,19,20^ and ensemble studies^21,22^, indicating that interaction with water vapour is a key determinant in impacts of varying viscosity on aerosol growth and evaporation, certainly within the atmospheric boundary layer, with consequences for particle size distributions, mass concentrations, air quality and climate (Fig. 1d)^8,11^. The generalisation of these results to regional or global scale processes relies on parameterisations that remain to be implemented^23^; pragmatically, viscous effects are often neglected in geographical regions dominated by biogenic sources of organic aerosol and high RH, for reasons we will see later.

Another area where knowledge of the viscosity of atmospheric OA is critical to understanding their environmental impacts is ice nucleation (Fig. 1b). Ice-nucleating particles (INPs) are extremely rare, and the abundance and temperature at which they nucleate ice are particularly important for understanding the formation and evolution of ice crystals in glaciated clouds^24^ and in turn global climate and precipitation. Unlike liquid particles that can only freeze homogeneously at a temperature determined by their water activity^25^, glassy organic aerosol can also nucleate ice heterogeneously via deposition nucleation or via immersion freezing on partially liquefied particles that undergo a kinetic transition from glassy to liquid^26,27^. Thus, highly viscous or glassy particles may add to the rare pool of atmospheric INPs, with the deposition or immersion pathways nucleating ice before reaching the homogeneous freezing limit. Ignatius et al.^28^ concisely summarise the viscous aerosol types which might add to the atmospheric INP pool. Laboratory studies have examined surrogates of atmospheric organic aerosol including pure citric acid^29^, sucrose^30^, raffinose^31^, levoglucosan^31^ and SOA particles formed by condensation of the low-volatility products from the oxidation of gas-phase volatile organic compounds (VOCs) such as naphthalene^27,32^ and α-pinene^28,33^.

The rates of heterogeneous reactions and photochemistry are also dependent on ambient particle viscosity (e.g. Fig. 1c). For example, gas-phase species, such as the hydroxyl radical (OH), ozone (O_3_) and the nitrate radical (NO_3_), continuously oxidise organic particles in the atmosphere. The viscosity of organic particles can impact the rates of these oxidation reactions, with implications for predicting the chemical composition and hygroscopicity^17,34–37^. The rates of uptake of other important gas-phase species (such as NH_3_, HO_2_, HCl, N_2_O_5_ and amines) also depend on the viscosity of organic particles, with important implications for predictions of atmospheric chemistry^38,39^. The growth of organic particles can occur by condensed-phase reactions of semi-VOCs; the rates of these reactions can be limited by slow bulk diffusion within a particle, and hence particle viscosity^40^. Solar radiation can photodegrade some of the components within atmospheric organic particles; viscosity has been shown to influence these photodegradation mechanisms, with higher viscosities leading to slower degradation rates^41,42^.

In the following section we review the approaches available for measuring the viscosity of aerosols particles. We then assess both laboratory and field measurements of aerosol particle viscosity and consider more fully the impact of phase on the properties of ambient aerosol. Finally, we consider the open questions that remain and the prospects of more completely representing the phase state of aerosols in regional and climate models.

## Direct, indirect and predictive methods for assessing aerosol viscosity

**Fig. 2:**
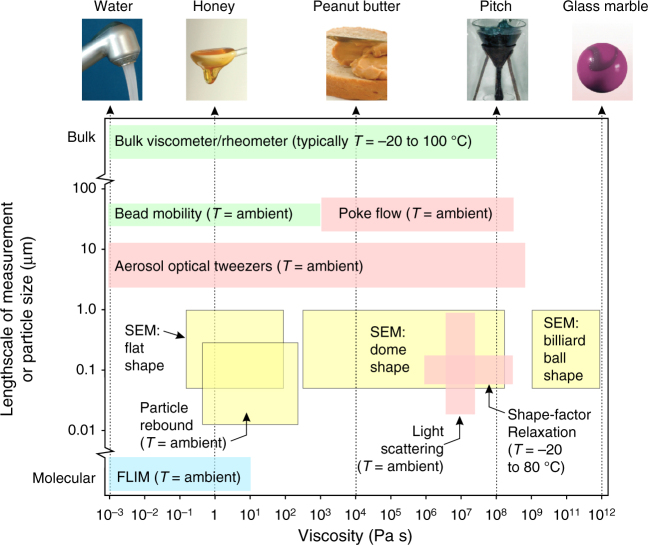
Size and viscosity ranges of techniques used to measure the viscosity of aerosol particles. Techniques dependent on resistance to an applied force, coalescence and shape relaxation, impaction on substrates or the use of molecular fluorescence probes are indicated by green, red, yellow and blue boxes, respectively. Where relevant, the temperature (*T*) range accessible to the technique is indicated. The top row indicates substances that have a viscosity typical of the value identified by the arrow from the bottom axis. SEM indicates the use of scanning electron microscopy and FLIM is fluorescence lifetime imaging microscopy. (Reproduced in part from ref. ^7^ with permission from the Royal Society of Chemistry)

Laboratory studies first suggested the possibility that ambient OA could exist as ultra-viscous or even glassy particles^5,6^. Observations that a fraction of ambient biogenic organic aerosol rebound on striking an impactor stage provided the first evidence that particles smaller than 100 nm could be highly viscous semi-solids or amorphous solids^4^. Following these pivotal publications, a number of experimental approaches have been developed to infer particle viscosity and to investigate the dependence on environmental conditions and particle composition. As identified in Fig. 2, these approaches are complementary in their capabilities accessing different viscosity ranges, particle sizes and compositions, and requiring different sample volumes. We describe some of these developments in capability and summarise some of the limitations in Table 1.

**Table 1:** Comparison of aerosol viscosity measurement techniques and capabilities

Technique	Viscosity range (Pa s)	Particle diameter range (μm)	Temperature range (°C)	Notes	References
Fluorescence lifetime imaging (FLIM)	10^−3^–10^3^	0.5–100	Ambient	*T* range can be extended by using a cooled/heated microscope stage. The upper viscosity limit could be extended using different molecular rotors	^35,43–45^
Particle rebound	10^0^–10^2^	<0.4	Ambient	RH dependence of rebound fraction measured to identify moisture content that gives rise to viscosity range specified	^46,47^
Dimer relaxation	5 × 10^5^–2 × 10^7^	~0.05–0.2	−15 to +80 °C	Identify *T*/RH conditions for transition in viscosity. Extrapolation to find *T*_g_ possible. Exact size and temperature ranges depend on many parameters	^48,49^
Light scattering	~10^7^	~0.1–0.9	−38 to +10 °C	Transition in depolarisation ratio (minimum size limit specified) from non-spherical to spherical shape on measurement timescale occurs at the viscosity indicated	^50^
Shape relaxation	10^5^–10^11^	<0.2	Ambient	Combination of particle diameter and mass measurements used to estimate shape factor	^51^
SEM imaging	10^−1^–10^12^	0.3–2	Ambient	Qualitative distinction between ‘solid’, ‘semi-solid’ and ‘liquid’ particles for complex field and laboratory-generated samples	^52^
Aerosol optical tweezers	10^−3^–10^9^	5–20	Ambient	Requires mL of sample. Measurable coalescence timescales span 10^−6^ to 10^5^ s. RH dependence of viscosity	^53–56^
Bead mobility	10^−3^–10^3^	30–50	Ambient	Samples collected with impactors can be analysed. RH dependence on viscosity can be determined	^57^
Poke flow	10^3^–10^7^	25–75	Ambient	Samples collected with impactors can be analysed. Uncertainties in the measured viscosities are large due to assumptions used when determine viscosity	^58,59^
Bulk viscometry/rheology	10^−3^–10^8^ typical	Bulk	−40 to 200 ^o^C typical	Crossover in storage and loss modulus allows identification of any phase state change	^60^

SEM, scanning electron microscopy; RH, relative humidity; *T*, temperature; *T*_*g*_, glass transition temperature

### Resistance to an applied force

The first class of techniques infers viscosity from measuring the resistance to an applied force. Although necessarily limited to large sample volumes, bulk phase measurements of viscosity are the obvious place to begin. Rheometer design varies according to the range of viscosity of interest^61^ and typically involves measurements of the radial displacement, torque and lateral force applied to a sample between the plates^53,60^. The lower plate often has a Peltier element allowing routine measurement of temperature dependence over a typical range of −20 to +100 ^o^C. The upper plate is moved sinusoidally back and forth and the response of the sample is decomposed into components in phase with the driving force (the storage modulus) and out-of-phase (the loss modulus). The viscosity is calculated from the ratio of the loss modulus to the oscillation frequency. As a bulk technique, there is a possibility of ‘forcing’ a phase state in systems where a metastable solution would instead persist in an aerosol droplet^53^. Such changes can be detected using the crossover in the storage and loss modulus to indicate where solid-like properties might begin to dominate over liquid-like properties^60^. Such changes are typically confirmed by other methods including X-ray diffraction and differential scanning calorimetry.

**Fig. 3:**
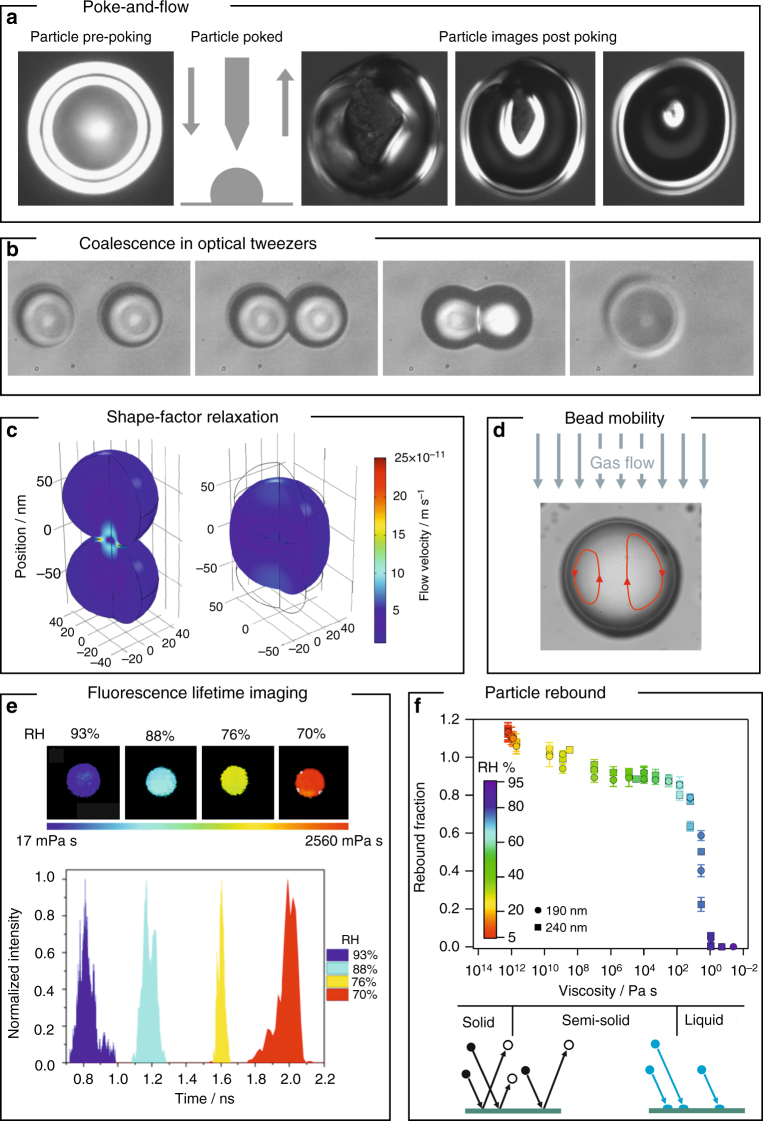
Summary of experimental techniques. **a** The relaxation in shape of a viscous droplet poked with a needle can be used to estimate viscosity in poke-and-flow experiments (images are a top view, schematic represents a side view). **b** The timescale for the relaxation in shape of two droplets coalescing in a holographic optical tweezer can be used to infer viscosity. **c** Simulation of the internal flow velocity during dimer coalescence/sintering following the coalescence of two particles as the composite particle approaches a spherical shape (reproduced from ref. ^51^).** d** Schematic illustrating the internal flow patterns in a particle that are used in the bead-mobility technique. **e** Example distributions of the fluorescence lifetimes of molecular rotors within sucrose droplets with varying relative humidity (RH) (reproduced from ref. ^45^). **f** Change in rebound fraction with viscosity for sucrose particles of two diameters (190 and 240 nm). The RH of the measurement (colour scale) is used to infer the viscosity (adapted with permission from ref. ^47^. Copyright (2015) American Chemical Society)

At the microscale, the bead-mobility technique can be used to measure the viscosity of realistic surrogates of ambient organic aerosol^57,58^. Melamine beads, 1 μm in diameter, are incorporated in large particles (30–50 μm diameter) formed from an organic aerosol sample deposited on a hydrophobic substrate. A shear stress is applied to the particle surface by a flowing gas and the melamine beads circulate in a well-defined pattern following the fluid flow (Fig. 3d). Bead positions are monitored with an optical microscope and the viscosity of the host particle determined from the velocity of the beads and a calibration curve that relates bead speed to viscosity. This technique is capable of inferring viscosities up to 10^3^ Pa s and metastable super-saturated states with respect to solutes can be accessed with reduced chance of forcing the phase state.

### Coalescence and shape relaxation

In addition to applying an external force, aerosol viscosities can be inferred by examining the interplay of the intrinsic inertial and viscous forces governing particle shape which force relaxation to a geometry that minimises surface free energy. Approaches of this type are appropriately limited to small volumes and have allowed measurements directly on suspended particles.

The poke-and-flow technique described by Renbaum-Wolff et al.^58^ and further validated by Grayson et al.^59^ builds on the qualitative technique introduced by Murray et al.^62^ Particles, 25–70 μm in diameter, are deposited on a hydrophobic substrate within a flow cell. While monitoring with an optical microscope, the particles are poked with a needle attached to a micromanipulator, leading to a non-equilibrium geometry that relaxes to minimise the surface energy of the system (Fig. 3a). An experimental flow time (related to the time required for the material to relax back to the equilibrium state) is determined and the viscosity inferred by comparison with simulated flow times. Viscosities ≥10^3 ^Pa s can be determined for particles with sub- and super-saturated solute concentrations.

A number of approaches have originated from monitoring the shape relaxation that occurs once particles coalesce. Using optical tweezers to capture pairs of aerosol particles (diameter 6–20 μm) in two optical traps, the coalescence event can be initiated at a well-defined time allowing accurate determination of the timescale for the capillary driven relaxation to a sphere (Fig. 3b)^53,54,56^. The final particle size and refractive index (thereby density) are determined from the wavelengths of resonant modes within the Raman spectrum and by comparison with Mie theory. For particles of low viscosity, below a critical damping threshold of ~0.02 Pa s, both the droplet surface tension and viscosity can be inferred^15,56^; at higher viscosity, a direct inverse relationship between viscosity and relaxation time allows the estimation of viscosities up to ~10^9^ Pa s, a wide dynamic range of ~12 orders of magnitude^53,56^. Similarly, inferring plausible timescales for shape relaxation of conjoined particles deposited on impactor stages (<100 nm diameter) can lead to constraints on the viscosity range of OA samples^63^.

Shape relaxation through sintering can also be observed indirectly by measuring the shape factor of suspended particles with diameters <200 nm. Here, the shape factor refers to a modification of the Stokes drag force experienced by a particle that travels with some velocity relative to its surrounding fluid. The shape factor is measured by comparing the sphere-equivalent volume diameter and apparent volume diameter in a mobility analyser. Particle agglomerates are passed for a set time through a conditioning tube at elevated *T* or RH. The observed change in shape factor is then related to viscosity using modified sintering theory (Fig. 3c)^48,49,51^. The accessible viscosity range depends on particle diameter and sintering time allowed, and is relatively narrow (10^5^–10^8^ Pa s) due to the challenges of working with very short (<5 s) or long (>20 min) residence times in continuous flow systems. A significant advantage is the capability to probe the thermodynamic conditions at which the transition from 10^7^ Pa s to 10^5^ Pa s occurs over a relatively wide range of temperature and relative humidities. Furthermore, the temperature dependence of viscosity in the semi-solid regime can be probed, and glass transition temperature can be obtained via extrapolation of temperature-dependent viscosity data to 10^12^ Pa s^48^. Particle dimers of any composition can be synthesised and isolated for analysis^64^, provided that sufficient particle number concentrations can be generated. To date, the technique has been applied to agglomerates that form from uncontrolled coagulation during the formation of SOA generated in a flow tube reactor^51^, as well as specially prepared and isolated dimers that form from coagulation of oppositely charged, single component particles that are prepared from atomised and dried aerosol^48,49^.

Complementing measurements of mobility shape factor, changes in the depolarisation ratio in light scattered from coagulated particles (>500 nm diameter) have been used to infer the degree of relaxation in shape and dependence on environmental conditions for SOA particles produced from α-pinene ozonolysis^50^. Measurements were performed in the CERN CLOUD chamber at temperatures covering the range −38 to 10 ^o^C. Following OA nucleation and growth, the depolarisation ratio was monitored with increasing RH. A transition range in RH was observed over which particles gained sufficient plasticising water to reduce the viscosity to a value <10^7^ Pa s, enabling relaxation to a spherical shape over the measurement timescale. As anticipated, the transition range in RH was found to increase as the temperature was reduced.

### Rebound from a rigid surface

As described above, observations of higher than anticipated rebound fractions for particles sampled over a boreal forest drew attention to the possible existence of amorphous solid-like ambient organic aerosol^4^. Submicrometer liquid droplets or other easily deformable particles tend to adhere to a substrate upon impaction, whereas solid or semi-solid particles tend to rebound. In practice, submicrometer atmospheric particles are accelerated through an orifice plate into high-velocity jets^46,65^. Upon impact with an underlying rigid surface, referred to as an impaction plate, particle rebound occurs when the in-going kinetic energy exceeds the at-impact energies of adhesion and dissipation (Fig. 3f)^46^. A low-viscosity particle can dissipate energy through many mechanisms, such as flattening, and thus does not rebound. By comparison, a highly viscous particle does not have these dissipation modes and thus rebounds. A size-dependent calibration is carried out using sucrose particles whose viscosity as a function of RH is known and varies by orders of magnitude^47^. The switchover from rebounding to non-rebounding particles occurs between 10^0^ and 10^2^ Pa s, corresponding to a transition to a liquid viscosity (Fig. 3f). The assumption is that other organic particles have a switch in rebound behaviour over a similar range of viscosity, yet scrutiny of this assumption is warranted given that physicochemical characteristics of the particle materials can influence energy relaxation upon impact^46,47^.

For particles that are deposited on a substrate, shape analysis can be used to provide qualitative information on viscosity by examining the particle deformation that occurs on impaction. In SEM images, liquid (10^−1^−10^2^ Pa s), semi-solid (10^2^−10^9^ Pa s) and solid (>10^9^ Pa  s) particles can be approximately separated based on height-to-base aspect ratios indicative of ‘flat’, ‘dome-like’ and ‘billiard ball’ shapes, respectively (see examples in Box 1, fig. a). The height-to-base ratios can be estimated directly from SEM images or from total absorption measurements of X-rays transmitted over equivalent projected areas of individual particles. The latter is performed using scanning transmission X-ray microscopy/near-edge X-ray absorption fine structure spectroscopy (STXM/NEXAFS), a chemical imaging technique capable of selective measurements over particles with similar chemical composition invaluable for identifying certain particle types within complex ensembles of ambient particles^52,66^. Among other factors, viscosity, surface tension, adhesion forces, contact angle and particle size influence the final shape in complex ways that cannot be easily quantified and correlated with precise viscosity values. Nevertheless, for particles collected on the same impactor stage, the height-to-base aspect ratios are a useful measure allowing qualitative comparison of viscosity between different particle types.

### Molecular probes

Fluorescence lifetime imaging (FLIM) exploits the viscosity dependent fluorescence lifetime of a group of molecules termed ‘molecular rotors’ to infer viscosities. The technique has a spatial resolution of ~250 nm, which allows imaging of supermicron objects, and has an extensive literature within the biomedical sciences, where it has been used to investigate intra-cellular viscosity in various organelles^67^. The choice of molecular rotor is dependent on the viscosity range being probed and other properties of the system, for example whether the system is hydrophilic or lipophilic. The volume probed by a molecular rotor is smaller than that probed by a translational diffusional viscosity probe. For homogeneous media, the rotational viscosity is the same as translational viscosity as shown by Dent et al.^68^ In heterogeneous media, the molecular rotor can preferentially partition between different micro-environments and, hence, measures the micro-viscosity, which can be different to the bulk translational viscosity. Changes in viscosity can be monitored in real time as the approach is non-destructive (Fig. 3e). Studies of deposited particles on microscope coverslips allow measurements on multiple particles simultaneously^43^. When combined with a counter-propagating optical trap, measurements can be made directly on suspended aerosol particles^45^. The molecular rotors are internally mixed in the sample at concentrations of tens of micromolar, sufficiently small to have negligible effect upon aerosol chemistry or microphysics. The technique has been used to measure the viscosity of simple aerosol systems such as single component and binary solution aerosols^43^ and complex systems such as SOA^35^, and to spatially resolve changes in viscosity during the oxidation of organic aerosol by both ozone and the hydroxyl radical^35,44^. On the microscale, it is well-known that droplet drying and reaction can lead to non-uniformities in composition and this technique is ideally placed to probe heterogeneities in viscosity^16,69^.

### Predictive models

A range of models is available to predict the viscosity of pure components^70^ and mixtures^71,72^. Group contribution methods, constrained by empirical data from a selection of known chemical compounds, provide an efficient framework for predicting the viscosity of a broader range of compounds for which data do not exist. Model performance is dependent on the range of species used in the fitting process and the ability to represent all structural features of a chosen molecule^73^. They have been shown to be accurate for compounds with viscosities <1 Pa s and capture the sensitivity of viscosity to the addition of successive functional groups^70,74^. Indeed, Song et al.^53^ confirmed that predictions were accurate up to viscosities of 10^4^ Pa s for pure component viscosities and from mixing rules for predicting RH-dependent trends for alcohol, di-, and tricarboxylic acid systems. They also reported that predictions overestimated the viscosity for the mono-, di-, and trisaccharide systems by many orders of magnitude, where subcooled viscosities of the pure components can exceed 10^12^ Pa s. Despite these recent studies, predictive methods remain largely unevaluated for systems exhibiting viscosities >1 Pa s and at temperatures approaching the glass transition^60^. It is likely that existing models are simply unconstrained for compounds relevant to atmospheric compositions, partly because the chemical characterisation of OA remains only partial. It is often unclear whether data represent measurements in the subcooled liquid state typical for ambient particles.

With the emergence of data from the techniques discussed in this report, there is potential to construct more accurate methods for use in atmospheric science. Given the rise of automated informatics software, it should become increasingly possible to supplement existing approaches to predict aerosol microphysics based on prescribed viscosity changes^18^ by mechanistic aerosol models that track the concentration of many thousands of compounds and predict particle properties directly. In addition, combinations of quantum chemical^75^ and thermodynamic models^76^ have the potential to support predictions of pure component viscosity^77^.

## Laboratory measurements of viscosity for particles of known composition

At typical aerosol concentrations observed in the atmosphere (between 0.1 and 100 μg m^−3^, the cumulative condensed phase mass per unit gas phase volume), it is not practical to acquire sufficient sample mass for all techniques that allow a quantitative determination of viscosity. In addition, the chemical composition is most often poorly constrained. Laboratory studies of aerosol particles of well-defined composition formed from readily available compounds can be used to test predictive models of viscosity, examining the influence of typical functional groups found in ambient particles, moisture content (RH dependence), temperature and molecular weight. From these “bottom-up” studies, “top-down” measurements of ambient aerosol particle phase and viscosity can be better understood and trends rationalised.

In the absence of a surface to catalyse heterogeneous nucleation, aerosol particles often exist as metastable subcooled liquids or amorphous glassy solids. Thus, to be representative of such metastable particle phases, measurements should be made in the aerosol phase rather than using bulk techniques. On cooling, molecular motions in the liquid phase can become sufficiently slow that the molecules are unable to equilibrate to the lowest energy state, a process often referred to as vitrification^78^. Below the glass transition temperature, *T*_g_, the sample behaves mechanically as a solid (viscosity >10^12^ Pa s), with the translational and re-orientational degrees of freedom of the molecules essentially frozen on the experimental timescale. Vitrification also occurs on drying, with aerosol particles able to pass through a moisture driven glass transition relative humidity, RH_g_. Conversely, water acts as a plasticiser: an increase in water activity in the condensed phase (RH in the gas phase) leads to increased molecular mobility and reduced viscosity. Thus, the dependence of aerosol particle viscosity on temperature and RH, two critical environmental quantities, must be understood and measurements are often reported with respect to one or both of these quantities.

**Fig. 4:**
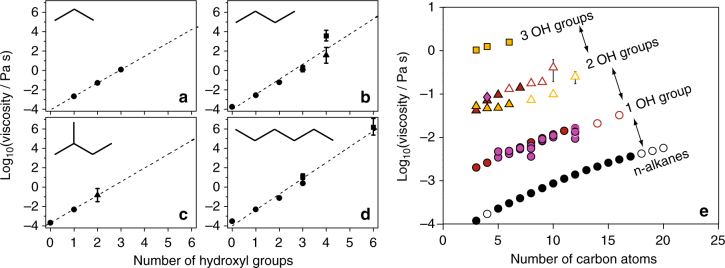
Dependence of viscosity on chemical composition. **a**–**d** Viscosity as a function of the number of hydroxyl functional groups for compounds with variable carbon backbone in dry conditions. Symbols: circles—literature data collated by Grayson et al.;^80^ squares—optical tweezers data from Song et al.;^53^ triangles: bead mobility^80^. The dashed lines are linear fits of all the available data. **e** Dependence of viscosity on the number of carbon atoms for compounds with a variable number of hydroxyl groups^74^. (Reprinted with permission from ref. ^74^. Copyright (2017) American Chemical Society)

For pure components, it is well-established that the glass transition temperature increases with the molecular weight (MW) of the compound (Fox-Flory relation)^7^. Further, it has been shown that the relationship between *T*_g_ and MW further stratifies with the atomic oxygen-to-carbon ratio, a commonly reported quantity for atmospheric aerosol compositions; a simple parameterisation for predicting *T*_g_ based on MW and O:C ratio has been developed^10^. Indeed, the dependence of viscosity on the identity of the chemical compound mirrors that of vapour pressure, i.e. a decrease in vapour pressure corresponds to an increase in viscosity, an observation that has been verified by some of the predictive tools described earlier^10,53,70,74,79^. For a limited range of functional groups, increases in viscosity through the systematic addition of functional units have been quantified^53,74,80^. For example, for compounds containing 2–6 hydroxyl groups, Song et al.^53^ (using optical tweezers) and Grayson et al.^80^ (using bead mobility and poke flow techniques) have observed an increase in the pure component viscosity by ~1–2 orders of magnitude for each additional hydroxyl group added to a carbon backbone (Fig. 4a–d). These measurements are consistent with a survey of bulk liquid phase literature data performed by Rothfuss and Petters for compounds containing between 0 and 3 hydroxyl at 298 K (Fig. 4e)^74^. More generally, the sensitivity of viscosity to functional group addition is: hydroxyl (C–OH) > nitrate (C–ONO_2_) > carbonyl (C = O) ≈ ester (O = COR) > methylene (CH_2_). Adding a first^74^ and then a second^53^ carboxyl group to a hydrocarbon chain (e.g. for the sequence pentane, pentanoic acid, glutaric acid) increases the viscosity stepwise by approximately one order of magnitude, comparable to the impact of the hydroxyl group. Adding a third carboxyl group and an OH group to form citric acid increases the viscosity by ~7 orders of magnitude, indicating considerable departure from simple additivity^53^. Further, ring structures increase viscosity relative to linear structures^74^.

The considerable impact of additional carboxyl and hydroxyl groups on the viscosity of organic compounds must be considered alongside the knowledge that these functional groups also promote hygroscopicity. For example, the viscosities of binary aqueous citric acid aerosol and aqueous 1,2,3,4,5,6-hexane-hexol (sorbitol) both fall by 9 orders of magnitude with increase in RH from 0 to 100% at 293 K^30^. However, the increasing glass transition temperature with MW is mirrored by a systematic increase in viscosity with progression from mono- to di- to tri-saccharides at a chosen RH^53,80^. Notably, the strong dependence of viscosity on RH for aqueous sucrose solutions, climbing from ~10^−3^ Pa s for dilute solutions to 10^12^ Pa s at ~25% RH^56^, has been used as a standard for calibrating a number of measurement techniques^43,47,57,59,81^. These dramatic changes with RH emphasise the importance of understanding the plasticising role of moisture. Indeed, the mixing state of inorganic and organic components is also crucial: the addition of sodium chloride to sucrose aerosol droplets has been shown to increase the moisture content at a specific RH, thereby reducing the viscosity by many orders of magnitude^56^. Thus, the aerosol hygroscopicity, quantified by the amount of water absorbed by the particle at a specified RH relative to the dry mass, strongly modulates the viscosity^47,48,53,56^. However, the viscosity of hygroscopic particles must approach that of water as the RH approaches 100% even for the most viscous of particles under dry conditions. This behaviour necessarily limits the impact of viscosity on kinetically determined processes such as the condensation of water and the activation of CCN to form cloud droplets at high RH^19,82^.

Atmospheric temperature typically varies between −70 and +40 °C. The strong decrease in viscosity with increasing temperature can be empirically described by a modified Arrhenius equation^83^. A decrease from 10^12^ Pa s (conventionally equated to that of a glass)^78^ to 10^6^ Pa s typically requires 20–30 K of warming beyond the glass transition temperature^48,74^. Viscosity decreases more gradually when warmed further. The non-linear response to temperature is self-evidently important when tracking the transport of particles through the atmosphere. Few measurement techniques are available to capture the temperature dependence of viscosity in the semi-solid regime. However, a combination of measurements of the glass transition temperature and temperature dependence in the semi-solid regime may ultimately prove sufficient to adequately constrain the temperature dependence for model aerosol systems.

The effect of particle size on viscosity is largely unknown. The majority of particles by number is confined to diameters <500 nm. As an intensive property, independent of sample size, the viscosities of aqueous sucrose particles of 10 µm and 100 nm size have been shown to be consistent in the semi-solid regime^48,56^. In addition, a number of studies indicate that *T*_g_ in particles is not significantly different from the bulk *T*_g_ when the particle size is greater than approximately 0.1 µm. This evidence includes thermodynamic arguments (based on the Kelvin equation)^84^ and numerous experimental studies (e.g. see refs. ^85,86^). However, Cheng et al.^84^ proposed that solid particles liquefy below a critical diameter, typically ~20 nm, that is inversely related to the temperature of the bulk phase transition. Thus, understanding the size dependence of viscosity is particularly important for understanding new particle formation^23^.

## Viscosity of complex organic aerosol from the laboratory to ambient measurements

We focus our attention in this section on laboratory and field measurements of the viscosity of SOA. The properties of primary atmospheric solid organic particles are discussed in Box 1.

### Laboratory-generated secondary organic aerosol

To establish the viscosity of SOA, laboratory-based environmental chambers or flow reactors have been used to generate SOA and the viscosity of the material generated measured using some of the techniques described above. In combination, several laboratory studies have shown that the viscosity of SOA depends on the VOC and oxidant used to generate the SOA^47,63,87–89^. For example, studies have revealed the following trend of decreasing viscosity with the VOC precursor under dry conditions: SOA from photooxidation of toluene ≥ SOA from ozonolysis of α-pinene > SOA from photooxidation of isoprene^87–89^. This trend is likely related to the degree of oxidation and average molecular weight of the SOA material produced^89^. Previously, laboratory studies have shown that the viscosity of many types of SOA is greater than the viscosity of peanut butter (~10^3^ Pa s) under dry conditions, and can even be greater than tar pitch (10^8^ Pa s) for some types of SOA^51,63,88,90^.

**Fig. 5:**
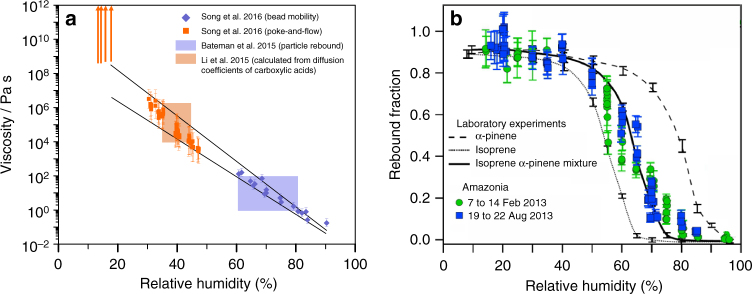
Viscosity and rebound fraction of secondary organic aerosols (SOA) as a function of relative humidity (RH). **a** Viscosity of toluene SOA as a function of RH determined with bead mobility, poke-and-flow, diffusion and particles rebound measurements^47,88^. Solid lines represent linear fits of the upper and lower boundaries of viscosity. **b** Rebound fraction of ambient SOA in Amazonia compared with measurements of laboratory-generated SOA^9^. (Reprinted by permission from Springer Nature Geoscience, ref. ^9^, 2016)

Consistent with the measurements of viscosity for aerosols with well-defined composition and the known plasticising effect of water, laboratory studies have shown that the viscosity of SOA depends strongly on the water activity (and hence water content) in the SOA^22,35,47,58,89^. As an example, the viscosity of SOA derived from the photooxidation of toluene increases by at least nine orders of magnitude as the water activity decreases from 0.9 to 0.2 (Fig. 5a)^88^. For some types of SOA, the viscosity has been shown to increase with the extent of oxidation^89^, consistent with other studies that have shown that the viscosity of organic aerosol increases when reacted with gas phase oxidants^44^. The presence of inorganic species also influences the viscosity of SOA. For example, the presence of ammonium sulfate decreases the viscosity of SOA generated in simulated cloud droplets, and the presence of sulfuric acid decreases the viscosity of SOA generated from the photooxidation of longifolene^89,91^. These results are important because ambient SOA is often found internally mixed with inorganics species, such as sulfates.

Experimental conditions of SOA generation, even when using the same oxidant and precursor VOC, also influence viscosity. For example, the RH at which the SOA is formed and the mass concentration of VOC used when generating the SOA can influence the viscosity of α-pinene SOA^81,92^. These results need to be considered when extrapolating laboratory results to atmospheric conditions, especially considering that VOC mass concentrations are frequently higher in laboratory studies and that the RH range studied is often limited. Viscosity has also been shown to vary as a function of particle size with plant chamber SOA appearing more liquid like at diameters < 30 nm and more solid like at diameters >30 nm^4,93^ consistent with previous reports^84^. Viscosity has also been found to increase as the more volatile components evaporate from an SOA sample^22^.

### Ambient organic aerosol

Due to the small amount of particulate material that can be collected from the atmosphere in a reasonable amount of time, studies related to the viscosity of ambient organic aerosol have relied on particle rebound experiments and SEM imaging. Several studies have focused on the viscosity (or related properties) of ambient biogenic SOA. The first studies reported were made in the boreal forest in the spring 2009 (Hyytiälä, Finland), a region strongly influenced by SOA from terpenes such as α-pinene^4^. During this field campaign, SOA was found to be non-liquid (>10^2^ Pa s) at ≤30% RH.

Field measurements have also been performed in the Amazon rain forest, a region strongly influenced by SOA from isoprene^9,94^. Based on rebound measurements, particles were liquid (<10^2^ Pa s) at RH >80% and 296–300 K during both the dry and wet seasons and periods not influenced by anthropogenic emissions (Fig. 5b). Dominated by these conditions much of the time, biogenic SOA particles are likely mostly in the liquid phase in the Amazon rain forest. Chemical images of biogenic SOA particles collected over the Amazon rain forest are also consistent with this conclusion^95^.

Researchers also carried out field measurements during the summer 2013 at a rural site in the south-eastern USA, a region dominated by SOA from isoprene and terpenes^96^. Organic-dominated particles were mostly liquid (<10^2^ Pa s) for typical RH and temperature values during the field campaign (RH > 70% and *T* > 295 K). On the other hand, at RH < 50% (which rarely occurred during the field campaign) the organic-dominated particles were often non-liquid (>10^2^ Pa s), consistent with expectations from laboratory measurements that have shown the plasticising influence of water on SOA viscosity.

The consequences of anthropogenic influences on the viscosity (or related properties) of ambient organic aerosol were observed during field measurements in the Amazon rain forest, with the rebound fraction greatly increasing at times of anthropogenic influence, indicative of semi-solid or solid particles^94^. Based on chemical imaging, the viscosity of ambient anthropogenic organic particles collected in various regions of North and South America was also found to be larger than the viscosity of biogenic SOA generated in the laboratory^52^. These combined results demonstrate the susceptibility of particle viscosity to anthropogenic perturbation.

## Concluding assessment and outlook

Currently, our understanding of the phase behaviour of ambient particles is largely underpinned by laboratory studies of atmospheric aerosol surrogates, apart from a few limited field measurements. Motivated by some of the early indications that organic aerosol particles can be glassy^4,6^, a number of complementary techniques have been developed to probe the viscosity of aerosol particles over a period of less than 10 years, either as samples deposited on substrates or as airborne particles. Some techniques (for example, aerosol optical tweezers) have allowed quantitative measurements of viscosity of airborne particles, but have proved unable as yet to facilitate measurements directly on ambient sub-micrometre organic particles. Other techniques have required calibration or allow identification of particles at threshold viscosities between two regimes (e.g., rebound measurements or measurements of shape factors to infer the progress of sintering). Some techniques have required measurements of samples on substrates, providing some of the first measurements of SOA samples collected in laboratory chamber studies (e.g., poke flow and FLIM). No one technique is sufficiently versatile to address the full range of particle sizes, compositions, viscosities, and sample sizes (ambient to bulk solutions). However, when all of these approaches are considered in combination, measurements of viscosities over an extremely large range spanning from 10^−3^ to >10^10^ Pa s can now be made, often with extremely small sample volumes and often avoiding crystallisation, essential when metastable highly viscous or glassy states must be understood. In addition, measurements have been made from −38 to +80 °C.

Using these techniques, studies have provided important information on the underlying chemical characteristics required for particles to exhibit high viscosities and they have mapped out the plasticising influence of water. These studies provide the molecular insight that can then allow the development of models of the physical properties of atmospheric particles (see, e.g., predictions of the viscosity of the components of oxidation of α-pinene organic aerosol in ref. ^53^). In addition, these techniques have identified the VOC precursors that are most likely to lead to viscous particles in the atmosphere through measurements on surrogates of ambient particles. In combination with a limited number of measurements of diffusion constants, laboratory measurements of viscosity have been crucial in confirming that the Stokes–Einstein equation provides a lower limit for values of the diffusion constants of diffusing species within a particle, allowing modellers to set an upper limit on the equilibration time of atmospheric aerosol particles. The experience gained from using these instruments (specifically those measuring rebound) for well-defined systems in the laboratory has allowed the same instruments to be confidently applied in a limited number of field measurements, providing insights into the viscosity of actual atmospheric aerosols. In addition, many of these tools show considerable promise for understanding the behaviour of viscous aerosol in a wider context, extending beyond the atmosphere^97^.

Some general conclusion about the phase state of organic aerosol in the atmosphere can now be made. Although primary organic particles emitted in certain geographical locations can be glassy, field measurements mostly suggest that biogenic SOA particles can be assumed to be liquid or semi-solid under the most prevalent environmental conditions in the planetary boundary layer, largely a consequence of the plasticising influence of water. The phase state of SOA originating from anthropogenic sources is more ambiguous with suggestions that particle viscosities could be considerably higher. These conclusions from field studies are broadly consistent with predictions of the global distribution of particle phase state^10,11^. Estimates of glass transition temperatures, based on the molar mass and molecular O:C ratio of SOA, suggest that SOA can be expected to be liquid in tropical and polar regions, semi-solid in mid-latitudes and solid over arid areas in the planetary boundary layer^10,11^. Conversely, SOA could exist in a glassy state in the middle and upper troposphere. With these limitations on particle viscosity and from Eqs. (1) and (2), SOA particles can be assumed to be well-mixed on timescales ≪1 h in the boundary layer. However, over arid regions and at higher altitudes, mixing timescales may exceed 1 day, imposing significant kinetic limitations on particle composition. This assessment suggests that much work remains in characterising the properties of SOA from anthropogenic precursor VOCs and the influence of temperature on the viscosity and glass transition temperature of SOA. More generally, the influence of temperature on the viscosity of benchmark organic aerosols and SOA surrogates remains largely unexplored.

Our understanding of the phase state of ambient OA has benefited considerably from the concerted approaches adopted in the laboratory, through field measurements and from modelling. To continue to clarify the prevalence and impacts of semi-solid and amorphous OAs on the atmosphere, these collaborations will continue to be important. There must continue to be a revolution in analytical techniques, with the capability to examine the necessarily small sample volumes available in ambient measurements and the ability to access a wide range in viscosity. Current techniques, notably SEM and rebound measurements, are limited to qualitative assessments of viscosity or measurements over a narrow viscosity range. The transition of laboratory techniques into the field is challenging given the low particle concentrations and large variations in environmental conditions over short timescales, but should be a priority. In addition, we have concentrated on aerosol particles that behave as Newtonian fluids in this review (i.e. fluids with a viscosity that is independent of shear or tensile stress). Although it is possible that aerosol particles can display behaviour typical of a non-Newtonian (nN) fluid (i.e. a fluid for which the viscosity depends on shear rate, e.g. polymer solutions, gels, liquid crystal phases)^5^, very little is known about the properties and behaviour of nN aerosol particles^98^. An important priority for future study will be to examine aerosol properties with techniques that are sensitive to the nN behaviour of particles, including aerosol optical tweezers and the particle rebound techniques.

To improve our understanding of atmospheric processes, refinements to predictive tools of aerosol microphysics is crucial, particularly if physically realistic parameterisations are to be provided for models on a global scale. As with laboratory approaches^82^, there needs to be sustained collaboration between mechanistic and empirically driven model developers across the community. Predictions of the viscosity of complex multicomponent mixtures must be tested, the accuracy of group contribution methods for predicting viscosity improved and the role of mixing state more fully established. For example, internal mixtures of inorganic components in organic aerosol can be expected to have a significant impact on viscosity while external mixtures may consist of population sub-sets of viscous/solid particles and liquid particles. The impact of heterogeneities in viscosity within a single particle, for example in phase-separated particles, remains unexplored. In laboratory surrogates of SOA, the impacts of environmental conditions (RH and temperature) and precursor concentration on particle viscosity remain poorly constrained. In the field, improvements must be made in identifying the critical chemical signatures of high viscosity, requiring advances in chemical characterisation tools. Indeed, simple predictive frameworks for viscosity based on quantities such as the O:C ratio may be insufficiently flexible.

Uncertainties in our knowledge of the phase state and viscosity of OA have consequences for understanding atmospheric aerosol processes. In particular, understanding phase state and viscosity is crucial for predicting the partitioning of organic components between the gas and particle phases, heterogeneous reaction rates of pollutants and the efficiency of ice nucleation. Although the viscosity is frequently used in conjunction with the Stokes–Einstein equation to estimate molecular diffusion constants and aerosol mixing timescales, it must be remembered that the validity of this approximation is sometimes acceptable^99^, but often unjustifiable^19,20,36,56^. Laboratory measurements on well-defined systems will continue to be important for testing the applicably of the Stokes–Einstein equation and determining if corrections can easily be made. Although a limited number of aerosol studies have reported diffusion constants for water in binary mixtures^19,20,69,100,101^, there are very few measurements of diffusion constants for organic compounds in aerosol particles of atmospherically relevant compositions^99,102,103^. These measurements are considerably more challenging to achieve and have never been performed with ambient aerosol samples. In addition, the interpretation of measurements relies on frameworks based on Fick’s laws, which must be extended to account for behaviour in highly complex mixtures. Instead, viscosity provides an important and tractable indicative property that can be used to set a lower limit on molecular diffusivity and, then, provides a route to assessing the global impacts of aerosols. Continued refinements to our understanding of aerosol particle phase state and viscosity are critical to achieve this goal.

### Box 1 Primary emitted solid (glassy) organic particles

Primary emitted solid organic particles occur in certain geographic areas where they can contribute substantially to local and regional atmospheric aerosols. Solid (glassy-like) tar balls are commonly reported as very abundant particles (up to 90%, by number) in the regions impacted by smoke from forest fires and from residential use of biomass fuels^104,105^. Tar balls are amorphous organic carbon particles with mean sizes of 100–300 nm and distinctive spherical morphology that does not deform upon impaction, indicative of their highly viscous state. They are ejected as liquid tar microdroplets from the pores of plants heated by fires and then solidify in the process of atmospheric aging^106^. Chemically similar to the tar balls, airborne soil organic particles (ASOP) were recently reported as a distinct particle type with their composition resembling soil organic matter^66^. Substantial contribution of ASOP (up to 60%, by number) was reported for the samples collected at the Southern Great Plains (Oklahoma, USA), and their emissions were attributed to atmosphere–land interactions during rainfall. These particles are released by bubble bursting from aqueous layers over wet soils after rain or irrigation events and, therefore, may be prevalent over grassland areas and irrigated agricultural fields. Despite their different emission mechanisms, both tar balls and ASOP contain large plant-derived degradation products, such as polysaccharides, tannins and lignin fragments, which have molecular structures substantially larger and less saturated than those characteristic for SOA. At the emission source, tar balls and ASOP exist as a liquid-like mist of water-soluble organic compounds, while later evaporation of water results in their solidification, consistent with the glassy-like spherical shapes (panels a and b). The particles have visible coatings on their surfaces, indicating their surfaces promoted heterogeneous chemistry and condensation of secondary species. Transported aloft, these viscous soil-like organic particles would provide solid glass surfaces for heterogeneous ice nucleation in cirrus and mixed-phase clouds^107^. The atmospheric role of macromolecular organic compounds from decomposing vegetation as strong ice nuclei has been discussed since the early seventies^108^. However, their emission sources have been traditionally attributed to wind erosion of soils, while relevance of the tar balls and ASOP was suggested only recently^66^. Also, because of their water-soluble nature, tar balls and ASOP would also serve as cloud condensation nuclei at lower altitude relevant to the formation of warm clouds. Finally, the macromolecular content of tar balls and ASOP also entails their ‘brown carbon’ optical properties with important contribution to the absorption and scattering of solar and terrestrial radiation^109^.

In the marine environment, nano- and micro-sized viscous organic gels containing colloids and aggregates exuded by phytoplankton contribute substantially to primary sea spray aerosol ejected into the atmosphere by waves and by bubble bursting at the water–air interface (panel c)^110,111^. As inferred from atmospheric modelling, laboratory studies and field observation, these marine organic particles are likely the dominant atmospheric ice nuclei relevant to remote marine environments such as the Southern Ocean, North Pacific Ocean and North Atlantic Ocean^112^. Marine organic gels were also detected in cloud water, suggesting that they play additional role in the activation of microdroplets of warm clouds^110^. Presently, chemical composition, molecular-level variability of marine gels, the extent of their external and internal mixing with other sea spray constituents, as well as their physical properties such as phase state, hygroscopicity, optical properties, etc. are insufficiently understood and are a subject of extensive research^113^.

Microscopy images of primary emitted atmospheric solid organic particles. **a** Particle sample collected at Lamont, Oklahoma, USA. Color-coded SEM image taken at the 75° tilt angle of the substrate-deposited glassy soil organic particles (green ‘billiard balls’) contrasting liquid particles flattened upon impaction (blue ‘flats’). Arrows indicate liquid-like coating flowed down onto the substrate as solid particles were impacted onto the substrate. (Reprinted by permission from *Nature Geoscience*, ref. ^66^, 2016.) **b** Particle sample collected aboard research aircraft flying near Mexico city. TEM image of tar balls emitted by biomass burning. (Reprinted with permission from ref. ^114^. Copyright (2011) by the American Geophysical Union.) **c** Particle sample collected aboard research ship in the high Arctic Ocean. Confocal optical microscopy image highlights viscous marine microgels detected among other airborne particles. (Reprinted with permission from ref. ^110^, Copyright (2011) by the National Academy of Sciences).

## References

[1] Pöschl U, Shiraiwa M (2015). Multiphase chemistry at the atmosphere–biosphere interface influencing climate and public health in the anthropocene. Chem. Rev..

[2] Hallquist M (2009). The formation, properties and impact of secondary organic aerosol: current and emerging issues. Atmos. Chem. Phys..

[3] Shrivastava M (2017). Recent advances in understanding secondary organic aerosol: implications for global climate forcing. Rev. Geophys.

[4] Virtanen A (2010). An amorphous solid state of biogenic secondary organic aerosol particles. Nature.

[5] Mikhailov E (2009). Amorphous and crystalline aerosol particles interacting with water vapor: conceptual framework and experimental evidence for restructuring, phase transitions and kinetic limitations. Atmos. Chem. Phys..

[6] Zobrist B, Marcolli C, Pedernera DA, Koop T (2008). Do atmospheric aerosols form glasses?. Atmos. Chem. Phys..

[7] Koop T, Bookhold J, Shiraiwa M, Pöschl U, Poeschl U (2011). Glass transition and phase state of organic compounds: dependency on molecular properties and implications for secondary organic aerosols in the atmosphere. Phys. Chem. Chem. Phys..

[8] Shiraiwa M, Seinfeld JH (2012). Equilibration timescale of atmospheric secondary organic aerosol partitioning. Geophys. Res. Lett..

[9] Bateman AP (2016). Sub-micrometre particulate matter is primarily in liquid form over Amazon rain forest. Nat. Geosci..

[10] Shiraiwa M (2017). Global distribution of particle phase state in atmospheric secondary organic aerosols. Nat. Commun..

[11] Maclean, A. M. et al. Mixing times of organic molecules within secondary organic aerosol particles: a global planetary boundary layer perspective. *Atmos. Chem. Phys*. **17**, 13037–13048 (2017). ***The authors conclude that the mixing timescale for individual particles is mostly <1 hour for secondary organic aerosol particles in the planetary boundary layer and suggest an assumption of a well-mixed aerosol in chemical transport models is reasonable for biogenic SOA***.

[12] Liu PF (2016). Mass lability of atmospheric organic particulate matter. Proc. Natl. Acad. Sci..

[13] Shiraiwa M, Zuend AA, Bertram AK, Seinfeld JH (2013). Gas-particle partitioning of atmospheric aerosols: interplay of physical state, non-ideal mixing and morphology. Phys. Chem. Chem. Phys..

[14] Krieger UK, Marcolli C, Reid JP (2012). Exploring the complexity of aerosol particle properties and processes using single particle techniques. Chem. Soc. Rev..

[15] Power RM, Reid JP (2014). Probing the micro-rheological properties of aerosol particles using optical tweezers. Rep. Prog. Phys..

[16] Bones DL, Reid JP, Lienhard DM, Krieger UK (2012). Comparing the mechanism of water condensation and evaporation in glassy aerosol. Proc. Natl. Acad. Sci. USA.

[17] Shiraiwa M, Ammann M, Koop T, Poeschl U (2011). Gas uptake and chemical aging of semisolid organic aerosol particles. Proc. Natl. Acad. Sci. USA.

[18] O’Meara S, Topping D, McFiggans G (2016). The rate of equilibration of viscous aerosol particles. Atmos. Chem. Phys..

[19] Lienhard DM (2015). Viscous organic aerosol particles in the upper troposphere: diffusivity-controlled water uptake and ice nucleation?. Atmos. Chem. Phys..

[20] Price HC (2015). Water diffusion in atmospherically relevant α-pinene secondary organic material. Chem. Sci..

[21] Qing Y (2016). Mixing of secondary organic aerosols versus relative humidity. Proc. Natl. Acad. Sci..

[22] Yli-Juuti T (2017). Factors controlling the evaporation of secondary organic aerosol from α-pinene ozonolysis. Geophys. Res. Lett..

[23] Zaveri Ra, Easter RC, Shilling JE, Seinfeld JH (2014). Modeling kinetic partitioning of secondary organic aerosol and size distribution dynamics: representing effects of volatility, phase state, and particle-phase reaction. Atmos. Chem. Phys..

[24] DeMott PJ (2010). Predicting global atmospheric ice nuclei distributions and their impacts on climate. Proc. Natl. Acad. Sci..

[25] Koop T, Luo B, Tsias A, Peter T (2000). Water activity as the determinant for homogeneous ice nucleation in aqueous solutions. Nature.

[26] Berkemeier T, Shiraiwa M, Pöschl U, Koop T (2014). Competition between water uptake and ice nucleation by glassy organic aerosol particles. Atmos. Chem. Phys..

[27] Charnawskas JC (2017). Condensed-phase biogenic-anthropogenic interactions with implications for cold cloud formation. Faraday Discuss..

[28] Ignatius K (2016). Heterogeneous ice nucleation of viscous secondary organic aerosol produced from ozonolysis of α-pinene. Atmos. Chem. Phys..

[29] Murray BJ (2010). Heterogeneous nucleation of ice particles on glassy aerosols under cirrus conditions. Nat. Geosci..

[30] Baustian KJ (2013). State transformations and ice nucleation in amorphous (semi-)solid organic aerosol. Atmos. Chem. Phys..

[31] Wilson TW (2012). Glassy aerosols with a range of compositions nucleate ice heterogeneously at cirrus temperatures. Atmos. Chem. Phys..

[32] Wang B (2012). The deposition ice nucleation and immersion freezing potential of amorphous secondary organic aerosol: pathways for ice and mixed-phase cloud formation. J. Geophys. Res. Atmos..

[33] Wagner R (2017). Heterogeneous ice nucleation of α-pinene SOA particles before and after ice cloud processing. J. Geophys. Res. Atmos..

[34] Berkemeier T (2016). Ozone uptake on glassy, semi-solid and liquid organic matter and the role of reactive oxygen intermediates in atmospheric aerosol chemistry. Phys. Chem. Chem. Phys..

[35] Hosny NA (2016). Direct imaging of changes in aerosol particle viscosity upon hydration and chemical aging. Chem. Sci..

[36] Marshall FHFH (2016). Diffusion and reactivity in ultraviscous aerosol and the correlation with particle viscosity. Chem. Sci..

[37] Steimer SS (2015). Shikimic acid ozonolysis kinetics in the transition from liquid aqueous solution to highly viscous glass. Phys. Chem. Chem. Phys..

[38] Gržinić G, Bartels-Rausch T, Berkemeier T, Türler A, Ammann M (2015). Viscosity controls humidity dependence of N_2_O_5_ uptake to citric acid aerosol. Atmos. Chem. Phys..

[39] Wang B (2015). Reactivity of liquid and semisolid secondary organic carbon with chloride and nitrate in atmospheric aerosols. J. Phys. Chem. A.

[40] Shiraiwa M (2013). Size distribution dynamics reveal particle-phase chemistry in organic aerosol formation. Proc. Natl. Acad. Sci..

[41] Wong JPS, Zhou S, Abbatt JPD (2015). Changes in secondary organic aerosol composition and mass due to photolysis: relative humidity dependence. J. Phys. Chem. A.

[42] Lignell H, Hinks ML, Nizkorodov SA (2014). Exploring matrix effects on photochemistry of organic aerosols. Proc. Natl. Acad. Sci. USA.

[43] Hosny NA (2013). Fluorescent lifetime imaging of atmospheric aerosols: a direct probe of aerosol viscosity. Faraday Discuss..

[44] Athanasiadis T (2016). Dynamic viscosity mapping of the oxidation of squalene aerosol particles. Phys. Chem. Chem. Phys..

[45] Fitzgerald C (2016). Fluorescence lifetime imaging of optically levitated aerosol: a technique to quantitatively map the viscosity of suspended aerosol particles. Phys. Chem. Chem. Phys..

[46] Bateman AP, Belassein H, Martin ST (2013). Impactor apparatus for the study of particle rebound: relative humidity and capillary forces. Aerosol Sci. Technol..

[47] Bateman AP, Bertram AK, Martin ST (2015). Hygroscopic influence on the semisolid-to-liquid transition of secondary organic materials. J. Phys. Chem. A.

[48] Rothfuss NE, Petters MD (2017). Characterization of the temperature and humidity-dependent phase diagram of amorphous nanoscale organic aerosols. Phys. Chem. Chem. Phys..

[49] Rothfuss NE, Petters MD (2016). Coalescence-based assessment of aerosol phase state using dimers prepared through a dual-differential mobility analyzer technique. Aerosol Sci. Technol..

[50] Järvinen E (2016). Observation of viscosity transition in α-pinene secondary organic aerosol. Atmos. Chem. Phys..

[51] Zhang Y (2015). Changing shapes and implied viscosities of suspended submicron particles. Atmos. Chem. Phys..

[52] O’Brien RE (2014). Physical properties of ambient and laboratory-generated secondary organic aerosol. Geophys. Res. Lett..

[53] Song YC (2016). Measurements and predictions of binary component aerosol particle viscosity. J. Phys. Chem. A.

[54] Bzdek BR, Collard L, Sprittles JE, Hudson AJ, Reid JP (2016). Dynamic measurements and simulations of airborne picolitre-droplet coalescence in holographic optical tweezers. J. Chem. Phys..

[55] Bzdek BR, Power RM, Simpson SH, Reid JP, Royall CP (2016). Precise, contactless measurements of the surface tension of picolitre aerosol droplets. Chem. Sci..

[56] Power, R. M., Simpson, S. H., Reid, J. P. & Hudson, A. J. The transition from liquid to solid-like behaviour in ultrahigh viscosity aerosol particles. *Chem. Sci*. **4**, 2597–2604 (2013). ***The new experimental technique described in this paper showed that the viscosity of aerosol particles could be measured directly over a wide range of more than 12 orders of magnitude with varying moisture content***.

[57] Renbaum-Wolff L, Grayson JW, Bertram AK (2013). Technical Note: New methodology for measuring viscosities in small volumes characteristic of environmental chamber particle samples. Atmos. Chem. Phys..

[58] Renbaum-Wolff L (2013). Viscosity of a-pinene secondary organic material and implications for particle growth and reactivity. Proc. Natl. Acad. Sci..

[59] Grayson JW, Song M, Sellier M, Bertram AK (2015). Validation of the poke-flow technique combined with simulations of fluid flow for determining viscosities in samples with small volumes and high viscosities. Atmos. Meas. Tech..

[60] Booth AM, Murphy B, Riipinen I, Percival CJ, Topping DO (2014). Connecting bulk viscosity measurements to kinetic limitations on attaining equilibrium for a model aerosol composition. Environ. Sci. Technol..

[61] Läuger J, Stettin H (2010). Differences between stress and strain control in the non-linear behavior of complex fluids. Rheol. Acta.

[62] Murray BJ (2012). Glass formation and unusual hygroscopic growth of iodic acid solution droplets with relevance for iodine mediated particle formation in the marine boundary layer. Atmos. Chem. Phys..

[63] Pajunoja A (2014). Estimating the viscosity range of SOA particles based on their coalescence time. Aerosol Sci. Technol..

[64] Maisels A, Kruis FE, Fissan H, Rellinghaus B, Zähres H (2000). Synthesis of tailored composite nanoparticles in the gas phase. Appl. Phys. Lett..

[65] Saukko E, Kuuluvainen H, Virtanen A (2012). A method to resolve the phase state of aerosol particles. Atmos. Meas. Tech..

[66] Wang B (2016). Airborne soil organic particles generated by precipitation. Nat. Geosci..

[67] Kuimova MK (2012). Mapping viscosity in cells using molecular rotors. Phys. Chem. Chem. Phys..

[68] Dent MR (2015). Imaging phase separation in model lipid membranes through the use of BODIPY based molecular rotors. Phys. Chem. Chem. Phys..

[69] Zobrist B (2011). Ultra-slow water diffusion in aqueous sucrose glasses. Phys. Chem. Chem. Phys..

[70] Sastri SRS, Rao KK (1992). A new group contribution method for predicting viscosity of organic liquids. Chem. Eng. J..

[71] Bosse, D. *Diffusion, Viscosity, and Thermodynamics in Liquid Systems.* (Technical University of Kaiserslautern, Germany, 2005).

[72] Cao W, Knudsen K, Fredenslund A, Rasmussen P (1993). Group-contribution viscosity predictions of liquid mixtures using UNIFAC-VLE parameters. Ind. Eng. Chem. Res.

[73] Bilde M (2015). Saturation vapor pressures and transition enthalpies of low-volatility organic molecules of atmospheric relevance: from dicarboxylic acids to complex mixtures. Chem. Rev..

[74] Rothfuss NE, Petters MD (2017). Influence of functional groups on the viscosity of organic aerosol. Environ. Sci. Technol..

[75] Klamt A, Schüürmann G (1993). COSMO: a new approach to dielectric screening in solvents with explicit expressions for the screening energy and its gradient. J. Chem. Soc., Perkin Trans..

[76] Klamt A, Jonas V, Bürger T, Lohrenz JCW (1998). Refinement and parametrization of COSMO-RS. J. Phys. Chem. A.

[77] Kurtén T (2016). α-pinene sutoxidation products may not have extremely low saturation vapor pressures despite high O:C ratios. J. Phys. Chem. A.

[78] Debenedetti PG, Stillinger FH (2001). Supercooled liquids and the glass transition. Nature.

[79] Mitra SS (1954). Erratum: Relation between vapor pressure and viscosity of liquids. J. Chem. Phys..

[80] Grayson JW (2017). The effect of adding hydroxyl functional groups and increasing molar mass on the viscosity of organics relevant to secondary organic aerosols. Atmos. Chem. Phys..

[81] Kidd C, Perraud V, Wingen LM, Finlayson-Pitts BJ (2014). Integrating phase and composition of secondary organic aerosol from the ozonolysis of α-pinene. Proc. Natl. Acad. Sci. USA.

[82] Marsh A (2017). Accurate representations of the physicochemical properties of atmospheric aerosols: when are laboratory measurements of value?. Faraday Discuss..

[83] Fulcher G (1925). Analysis of recent measurements of the viscosity of glasses. J. Am. Ceram. Soc..

[84] Cheng Y, Su H, Koop T, Mikhailov E, Pöschl U (2015). Size dependence of phase transitions in aerosol nanoparticles. Nat. Commun..

[85] Alcoutlabi M, McKenna GB (2005). Effects of confinement on material behaviour at the nanometre size scale. J. Phys.-Condens. Matter.

[86] Mattsson J, Forrest JA, Börjesson L (2000). Quantifying glass transition behavior in ultrathin free-standing polymer films. Phys. Rev. E.

[87] Song M (2015). Relative humidity-dependent viscosities of isoprene-derived secondary organic material and atmospheric implications for isoprene-dominant forests. Atmos. Chem. Phys..

[88] Song M (2015). Relative humidity-dependent viscosity of secondary organic material from toluene photo-oxidation and possible implications for organic particulate matter over megacities. Atmos. Chem. Phys..

[89] Saukko E (2012). Humidity-dependent phase state of SOA particles from biogenic and anthropogenic precursors. Atmos. Chem. Phys..

[90] Abramson E, Imre D, Beránek J, Wilson J, Zelenyuk A (2013). Experimental determination of chemical diffusion within secondary organic aerosol particles. Phys. Chem. Chem. Phys..

[91] Schill GP, Tolbert MA (2014). Heterogeneous ice nucleation on simulated sea-spray aerosol using Raman microscopy. J. Phys. Chem. C.

[92] Grayson JW (2015). Effect of varying experimental conditions on the viscosity of α-pinene derived secondary organic material. Atmos. Chem. Phys..

[93] Virtanen A (2011). Bounce behavior of freshly nucleated biogenic secondary organic aerosol particles. Atmos. Chem. Phys..

[94] Bateman AP (2017). Anthropogenic influences on the physical state of submicron particulate matter over a tropical forest. Atmos. Chem. Phys..

[95] Pöschl U (2010). Rainforest aerosols as biogenic nuclei of clouds and precipitation in the Amazon. Science.

[96] Pajunoja A (2016). Phase state of ambient aerosol linked with water uptake and chemical aging in the southeastern US. Atmos. Chem. Phys..

[97] Baldelli A, Power RM, Miles REH, Reid JP, Vehring R (2016). Effect of crystallization kinetics on the properties of spray dried microparticles. Aerosol Sci. Technol..

[98] Pfrang C (2017). Complex three-dimensional self-assembly in proxies for atmospheric aerosols. Nat. Commun..

[99] Chenyakin Y (2017). Diffusion coefficients of organic molecules in sucrose-water solutions and comparison with Stokes-Einstein predictions. Atmos. Chem. Phys..

[100] Davies JF, Wilson KR (2016). Raman spectroscopy of isotopic water diffusion in ultra-viscous, glassy and gel states in aerosol using optical tweezers. Anal. Chem..

[101] Steimer SS (2015). Electrodynamic balance measurements of thermodynamic, kinetic, and optical aerosol properties inaccessible to bulk methods. Atmos. Meas. Tech..

[102] Bastelberger S, Krieger UK, Luo B, Peter T (2017). Diffusivity measurements of volatile organics in levitated viscous aerosol particles. Atmos. Chem. Phys..

[103] Price HC, Mattsson J, Murray BJ (2016). Sucrose diffusion in aqueous solution. Phys. Chem. Chem. Phys..

[104] Hand JL (2005). Optical, physical, and chemical properties of tar balls observed during the Yosemite Aerosol Characterization Study. J. Geophys. Res. Atmos..

[105] Pósfai M (2004). Atmospheric tar balls: particles from biomass and biofuel burning. J. Geophys. Res.

[106] Tóth A, Hoffer A, Nyiro-Kósa I, Pósfai M, Gelencsér A (2014). Atmospheric tar balls: aged primary droplets from biomass burning?. Atmos. Chem. Phys..

[107] Wang B, Knopf DA (2011). Heterogeneous ice nucleation on particles composed of humic-like substances impacted by O3. J. Geophys. Res. Atmos..

[108] Schnell RC, Vali G (1972). Atmospheric ice nuclei from decomposing vegetation. Nature.

[109] Alexander Wa, Zhang J, Murray VJ, Nathanson GM, Minton TK (2012). Kinematics and dynamics of atomic-beam scattering on liquid and self-assembled monolayer surfaces. Faraday Discuss..

[110] Orellana MV (2011). Marine microgels as a source of cloud condensation nuclei in the high Arctic. Proc. Natl. Acad. Sci..

[111] Aller JY (2017). Size-resolved characterization of the polysaccharidic and proteinaceous components of sea spray aerosol. Atmos. Environ..

[112] Wilson TW (2015). A marine biogenic source of atmospheric ice-nucleating particles. Nature.

[113] Cochran RE (2017). Molecular characterization of sea spray particles: influence of ocean biology on particle composition and interaction with water. Chem.

[114] Adachi K, Buseck PR (2011). Atmospheric tar balls from biomass burning in Mexico. J. Geophys. Res. Atmos..

